# Ori-Finder: A web-based system for finding *oriC*s in unannotated bacterial genomes

**DOI:** 10.1186/1471-2105-9-79

**Published:** 2008-02-01

**Authors:** Feng Gao, Chun-Ting Zhang

**Affiliations:** 1 Department of Physics, Tianjin University, Tianjin 300072, China

## Abstract

**Background:**

Chromosomal replication is the central event in the bacterial cell cycle. Identification of replication origins (*oriC*s) is necessary for almost all newly sequenced bacterial genomes. Given the increasing pace of genome sequencing, the current available software for predicting *oriC*s, however, still leaves much to be desired. Therefore, the increasing availability of genome sequences calls for improved software to identify *oriC*s in newly sequenced and unannotated bacterial genomes.

**Results:**

We have developed Ori-Finder, an online system for finding *oriC*s in bacterial genomes based on an integrated method comprising the analysis of base composition asymmetry using the *Z*-curve method, distribution of DnaA boxes, and the occurrence of genes frequently close to *oriC*s. The program can also deal with unannotated genome sequences by integrating the gene-finding program ZCURVE 1.02. Output of the predicted results is exported to an HTML report, which offers convenient views on the results in both graphical and tabular formats.

**Conclusion:**

A web-based system to predict replication origins of bacterial genomes has been presented here. Based on this system, *oriC *regions have been predicted for the bacterial genomes available in GenBank currently. It is hoped that Ori-Finder will become a useful tool for the identification and analysis of *oriC*s in both bacterial and archaeal genomes.

## Background

With the increasing availability of complete genome sequences, it has become clear that an essential factor influencing bacterial proliferation is the organization of the *oriC *region. In bacteria, chromosome replication initiates at a single chromosome locus, called the replication origin (*oriC*), from which replication proceeds bidirectionally to the terminus. At the beginning of replication, ATP binds DnaA, resulting in a large oligomeric complex consisting of DnaA monomers bound to a series of 9-mer consensus elements termed DnaA boxes [1]. As a general rule, bacterial chromosomes are characterized by one bidirectional origin of replication and one terminus that define the boundaries of replichores [2]. The replication process is asymmetric with respect to the two DNA strands from the fact that the leading strand is synthesized continuously, whereas the lagging strand is synthesized as Okazaki fragments. DNA strand asymmetry can also be described in terms of gene content and nucleotide composition. Moreover, all rRNA genes, the majority of ribosomal protein coding genes and essential genes are mainly transcribed in the same direction as DNA replication (leading strand) [2]. Therefore, identifying the *oriC*s of bacteria is one of the key steps not only in understanding the mechanisms of bacterial replication but also in gaining insight into gene distribution and function of these bacterial genomes.

The pioneer work to identify *oriC*s *in silico *is the GC-skew analysis, which is deemed as a routine method [3,4]. Based on the GC-skew analysis, Frank and Lobry also developed Oriloc, a program for predicting bacterial *oriC*s [5]. Meanwhile, an oligomer-skew method was also proposed to predict *oriC *regions in bacterial genomes [6], and later used to identify *oriC*s in 200 bacterial or/and archaeal genomes [7]. Use of GC-skew analysis together with location of the *dnaA *gene and distribution of DnaA boxes led to more accurate prediction of *oriC *regions [8].

Identification of *oriC*s is necessary for almost all newly sequenced bacterial genomes. Given the increasing pace of genome sequencing, the currently available software for predicting *oriC*s, however, still leaves much room to be improved. For instance, 1) Most, if not all, available software only predicts one approximate position instead of the boundaries of *oriC*s. Since the *oriC *is an intergenic region rather than one site, it is necessary to present *oriC *regions with definite boundaries. 2) Most software does not provide the distribution of species-specific DnaA boxes, which are strong indicators of *oriC*s. 3) No automated web server is available so far, e.g., Oriloc, in which the program can be downloaded and run locally by users. Web-based tools are more convenient, since they are platform independent and there is no need to download and install the software. 4) Only the GC-skew and/or AT-skew are provided. In addition to G/C and A/T bases, R/Y and M/K bases can also show asymmetrical distributions around *oriC*s. It is more informative to show all the base composition asymmetries than using GC and/or AT-skews alone.

To identify *oriC *regions of unannotated bacterial genomes, we have developed an online tool, Ori-Finder, based on an integrated method comprising gene identification, analysis of base composition asymmetry using the *Z*-curve method, distribution of DnaA boxes, occurrence of genes frequently close to *oriC*s and phylogenetic relationships. Output results that contain detailed information of predicted *oriC*s, DnaA box distributions, base composition disparity curves, and coordinates of identified genes by ZCURVE 1.02, are exported to an HTML report, which offers convenient views in both graphical and tabular formats.

## Implementation

The web server Ori-Finder is implemented on Apache server and the web interface is designed using CGI (Common Gateway Interface) Perl scripts. The algorithms to predict *oriC *regions of bacterial genomes *in silico *are complemented with the language of C++. The output graphs are generated by gnuplot graphic routine [9].

The software kit consists of the following programs.

(1) The program to calculate the coordinates of the RY, MK, GC and AT disparity curves [10].

(2) The program to calculate the distribution of DnaA boxes for the input genome sequence.

(3) The program to assign and rank the priority of every intergenic sequence.

(4) The gene-finding program ZCURVE 1.02 [11].

(5) The Perl script integrating the above programs into one system.

### Input to sever

Ori-Finder has a user-friendly and intuitive input interface. Users can choose to paste the sequence into the input box or upload the sequence (FASTA format) in a file. Additionally, the server requires the specification of some optional parameters listed as follows.

(1) Select 'species-specific' DnaA boxes according to the species. It defaults to the *Escherichia coli *perfect DnaA box (TTATCCACA). In addition, 'species-specific' DnaA boxes have also been provided, and some of them are proposed based on the phylogenetic relationships, which have not been reported previously, such as 'TTTTCCACA' for *Chlamydiae*, 'TTATCGAAA' for *Dehalococcoides *and 'TGTTTCACG' for *Bradyrhizobiaceae *etc. For example, the motif 'TGTTTCACG' is proposed to server as DnaA boxes for the genomes of the family *Bradyrhizobiaceae *(including *Rhodopseudomonas*, *Nitrobacter *and *Bradyrhizobium*). Currently, ten genomes of *Bradyrhizobiaceae *have been fully sequenced. Note that the predicted *oriC *regions of them are all closely next to *gidA *gene as well as the switch of GC disparity curve, and all contain the palindromic sequence 'TGTTTCACGTGAAACA', which is composed of two proposed DnaA boxes 'TGTTTCACG'. Consequently, the 'species-specific' DnaA boxes proposed by us are consistent with the phylogenetic relationships. Users can also choose to search for the DnaA boxes defined themselves. By default, only nonamers differing in no more than one position from the selected DnaA boxes are scanned, but this can be changed according to the requirements of users.

(2) Whether to upload a protein table file (".ptt" file) containing the coordinates of genes in the input genome sequence. The ".ptt" file is a table of protein features, which is usually provided by GenBank in NCBI's RefSeq ftp site [12]. Users can choose to download the ".ptt" file provided by GenBank or produce their own genome annotation that has the same format as the ".ptt" file according to their requirements. For Ori-Finder, if no ".ptt" file is uploaded, the gene-finding program ZCURVE 1.02 will be called to generate the gene list. In general, ZCURVE 1.02 or other gene-finding software alone couldn't annotate gene function. To obtain the ".ptt" file with annotation of genes related to *oriC*s, a database of indicator genes (such as *dnaA*, *dnaN*, *hemE*, *gidA *etc) has been collected by us, and BLAST program has also been installed locally for users' convenience (click 'Database' on the home page for more detail). Therefore, users can Blast the query sequences and find the homologues of these genes in the sequences.

(3) Whether to display RY, MK, GC or AT disparity curves and the DnaA box distribution in the output graphs. It defaults to displaying all of them in the output graphs.

### Output from the sever

**Figure 1:**
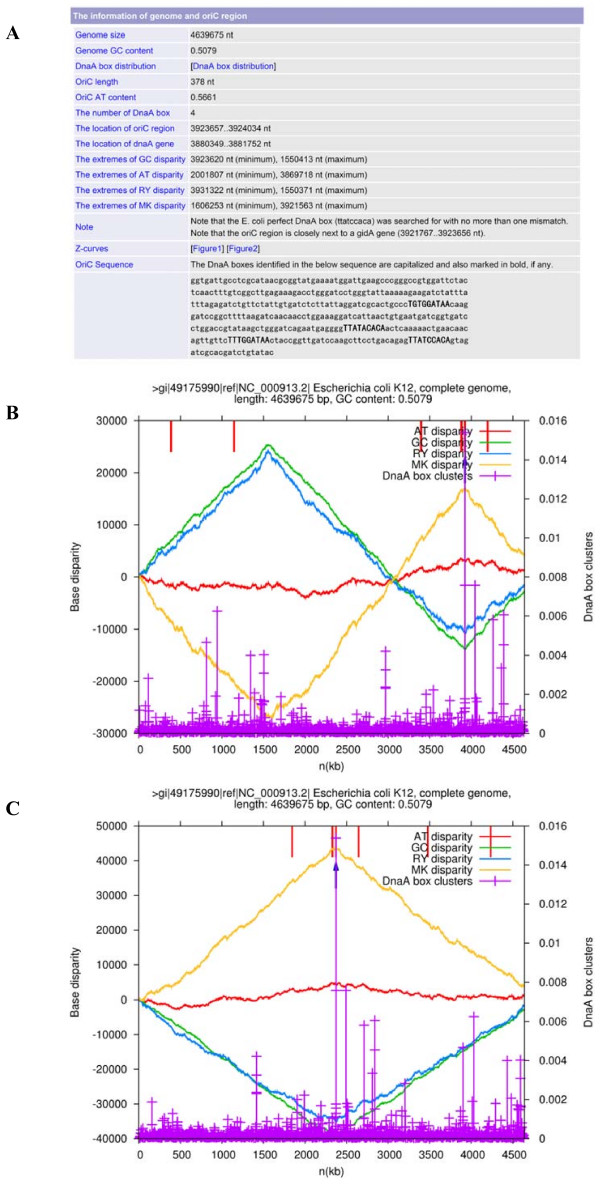
**A typical example of the output results obtained by Ori-Finder**. (A) The information of genome and *oriC *region for *Escherichia coli *K12 as an HTML table. (B) The integrated plot as a PNG figure for the original sequence and (C) the integrated plot as a PNG figure for the rotated sequence, displaying the obtained results, such as general genome information, four disparity curves, distribution of DnaA boxes, locations of putative indicator genes and the predicted *oriC *region. Note that the coordinate origin of the rotated sequence begins and ends in the maximum of the GC disparity curve.

**Figure 2:**
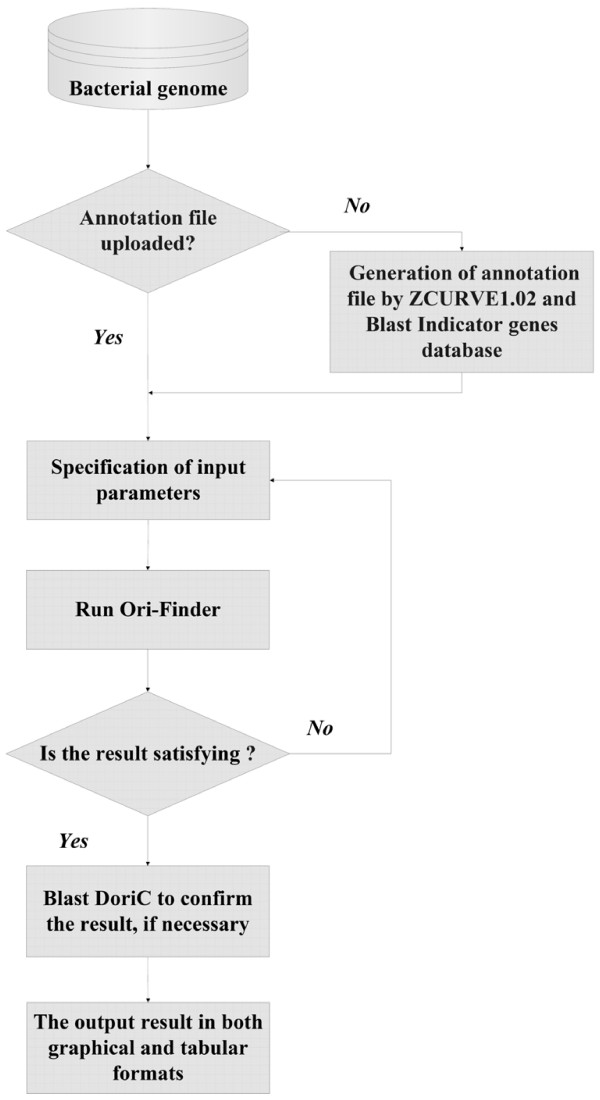
**The procedure of Ori-Finder to identify *oriC *regions**. Flow chart schematically showing the procedure to identify *oriC *regions by Ori-Finder.

The output web page shows the process of Ori-Finder, and provides links to the output results by Ori-Finder: (i) the genome size, GC content, location, length, DnaA box number, the motif of DnaA boxes, the conserved genes adjacent to the predicted *oriC*s, the precise coordinates of extremes of the four disparity curves and the sequence of identified *oriC *regions as an HTML table; (ii) the DnaA box distribution and the coordinates of the four disparity curves as text files; (iii) the integrated plot for the original sequence and (iv) that for the rotated sequence to display the obtained results, such as general genome information, the four disparity curves, distribution of DnaA boxes, locations of indicator genes and *oriC *regions in PNG format. A typical example of the output results obtained by Ori-Finder is shown in Figure 1. If no ".ptt" file is uploaded, the gene list file generated by ZCURVE1.02 will also be outputted. Users can do a Blast search using each output result by Ori-Finder against DoriC, a database of *oriC *regions [13], to confirm the reliability of the prediction. Refer to Figure 2 for the complete procedure of Ori-Finder to identify *oriC *regions. The output results by Ori-Finder for the newly sequenced genomes in GenBank are also available at our website.

## Results

Based on this online system, we have predicted *oriC *regions for the bacterial genomes available in GenBank currently. To estimate the accuracy of the predicted *oriC*s by Ori-Finder, a simple and straight way is to compare the predicted *oriC*s with those confirmed by experimental data. Consequently, the experimental confirmed *oriC*s so far are in accordance with those predicted by Ori-Finder [13]. In addition, other lines of evidence from *oriC *types, the coherence of all the three methods (i.e., typical base composition asymmetry, DnaA box distribution and indicator genes positions), and comparative genomic analysis, have shown the high reliability of the predicted *oriC*s by Ori-Finder.

In what follows we will discuss the unique merits of the present method, showing the evidence why this method is superior to other methods currently available. First of all, the *in silico *methods currently available to identify *oriC*s include the GC-skew analysis, and the oligomer-skew method etc. Jointly using the three methods (GC-skew, location of the *dnaA *gene and distribution of DnaA boxes) resulted in better prediction of *oriC *regions by Mackiewicz *et al *[8]. Although DnaA and its binding sites are well conserved throughout the bacterial kingdom, replication origins from different species show considerable diversity in terms of the number, arrangement and even the consensus sequence of DnaA boxes. In addition, genes adjacent to *oriC*s include not only *dnaA *gene itself but also *dnaN*, *hemE*, *gidA *or *hemB *etc. The information mentioned above has all been taken into consideration by the present approach, such as 'species-specific' DnaA boxes, possible genes adjacent to *oriC*s and phylogenetic relationships. However, by analyzing the GC-skew, Blasting for the *dnaA *gene and searching for DnaA boxes, the *oriC*s for only ~60% of genomes can be predicted. For example, the *oriC*s were identified for only 76 chromosomes out of the 120 chromosomes (76/120 = 63.3%) [8], which has also been confirmed again by our result for 568 chromosomes available currently in GenBank. Out of the 568 chromosomes, there are only 342 chromosomes (342/568 = 60.2%) whose *oriCs *are adjacent to *dnaA *gene and near the switch of GC-skew. Second, the present work can find *oriC *regions with definite boundaries with the accuracy of single base pair. Consequently, the integrated method presented in this paper outperforms those jointly applying the three methods mentioned above, as reflected by the fact that using the integrated method, the *oriC *regions can be identified for over 98% chromosomes out of the 568 bacterial chromosomes analyzed. For example, the genome of *Synechococcus *sp. JA-3-3Ab (NC_007775) does not exhibit any typical GC skew pattern. Therefore, based on the location of the *dnaA *gene, the origin is tentatively assigned to the *rpmH*-*dnaA *intergenic region [14]. However, in the family of *Synechococcus *genomes the *oriC *regions are closely adjacent to *dnaN *gene rather than *dnaA *gene and the DnaA box motif is 'TTTTCCACA' rather than 'TTATCCACA'. Here, by taking the above information into account, the *oriC *of *Synechococcus *sp. JA-3-3Ab can be easily predicted by Ori-Finder. For another example, applying the methods based on GC-skew, *dnaA *gene location and *E. coli *perfect DnaA box distribution to the genome of *Synechococcus *sp. WH 8102 (NC_005070), each of the three methods identifies a different region, respectively [8]. Consequently, none *oriC *can be identified for this genome [8], whereas a reliable *oriC *for this genome has been identified by the integrated method Ori-Finder.

Finally, we should point out the limitation of the present method. Note that the replication origins from different species show considerable diversity and sometimes annotation mistakes affect the performance. Therefore, the present method couldn't solve the problem of identifying the *oriC *for every bacterial genome currently available, of course, nor could other methods. Accordingly, *oriC*s for only ~2% of 568 chromosomes remain unresolved currently using the integrated method presented here, whereas ~40% of 568 genomes cannot be solved using only the information of GC-skew, location of the *dnaA *gene and distribution of DnaA boxes. However, for the chromosomes whose *oriC*s remain unresolved by Ori-Finder, the distribution of DnaA boxes and the extremes of GC (AT, RY and MK) disparity curves are still provided by Ori-Finder, which are helpful to obtain the approximate position of *oriC *region.

For the convenience of users' query, the predicted *oriC *regions have been organized into a MySQL database, called DoriC, which is freely available online [15].

## Conclusion

With the availability of an increasing number of bacterial genomes, the prediction will be more accurate and reliable since the DnaA boxes or genes frequently close to *oriC*s can be analyzed by comparative genomics. Currently, the web server Ori-Finder is designed only for the identification of *oriC*s in bacterial genomes. However, the architecture of the system is also suitable for archaeal genomes by changing DnaA box into ORB (origin recognition boxes) elements. The current version contains an input option by which users can define their own DnaA boxes or ORB elements. It is hoped that Ori-Finder will become a useful tool for the identification and analysis of *oriC*s in both bacterial and archaeal genomes.

## Availability and requirements

**Project name: **Ori-Finder

Project homepage: 

**Operating systems: **platform independent

**Programming language: **C++

**License: **Freely available

**Any restrictions on use by non-academics: **None

## Authors' contributions

FG designed the computer program and drafted the manuscript. CTZ supervised the study and revised the manuscript. Both authors read and approved the final manuscript.
